# Why do patients not return to sports or work after anatomical or reverse total shoulder arthroplasty? A systematic review and meta-analysis

**DOI:** 10.1016/j.jseint.2025.05.028

**Published:** 2025-06-07

**Authors:** Wouter J. van der Poel, Arno A. Macken, Denise Eygendaal, Geert A. Buijze, Michel P.J. van den Bekerom

**Affiliations:** a Department of Orthopaedics and Sports Medicine, Erasmus MC, Rotterdam, The Netherlands; b Department of Orthopaedic Surgery, Alps Surgery Institute, Annecy, France; c Department of Orthopaedic Surgery, Spaarne Gasthuis, Hoofddorp, The Netherlands; d Department of Orthopaedic Surgery, OLVG, Amsterdam, The Netherlands; e Department of Orthopaedic Surgery and Sports Medicine, Vrije Universiteit, Amsterdam, The Netherlands

**Keywords:** Reverse shoulder arthroplasty, Anatomical shoulder arthroplasty, Return to sport, Return to work, Meta-analysis, Systematic review, Shoulder

## Abstract

**Background:**

Several studies have been published regarding the rate of return to sports or work after shoulder arthroplasty. However, there are no systematic reviews regarding the reasons for not returning to sports or work. The aim of this study is to assess the rate of return to sports or work and the reasons not to return to sports or work in patients undergoing total shoulder arthroplasty.

**Methods:**

The search was performed on the 23rd of April 2024 in multiple databases. Studies reporting return to work or return to sport after anatomic total shoulder arthroplasty (aTSA) or reverse total shoulder arthroplasty (rTSA) with a minimum follow-up of 2 years and studies reporting the reasons for no return to sport or no return to work after anatomical or reverse shoulder arthroplasty were included.

**Results:**

Our search resulted in 393 articles, of which 31 studies were included. In total, 18 studies reported the reasons for not returning, whereas 13 did not. The mean (95% confidence interval) return rate to sport was 91% (86%-95%) after aTSA and 80% (72%-89%) after rTSA. Reasons not to return to sport were shoulder related in 13 of the 27 cases after aTSA and in 6 of the 19 cases after rTSA. The mean (95% confidence interval) return rate to work was 76% (59%-93%) after aTSA and 46% (26%-66%) after rTSA. Reasons not to return to work were shoulder related in 4 of the 15 cases after aTSA and in 8 of the 35 cases after rTSA.

**Conclusion:**

A high return to sport can be expected after total shoulder arthroplasty. The rate of return to work after aTSA is high; this is in contrast to patients with an rTSA who are less likely to return to work. Interestingly, most reasons not to return to sport or work after shoulder arthroplasty are not shoulder related. Reporting of reasons not to return to sport or work is limited; this should be considered in future studies.

Total shoulder arthroplasty (TSA), as anatomic total shoulder arthroplasty (aTSA) and reverse total shoulder arthroplasty (rTSA), has become a more popular procedure over the last few decades. A meta-analysis from 11 joint public registers showed an annual growth rate of 6%-15% between 2011 and 2019 of shoulder replacements performed.^36^ This increase can be attributed to an expansion of indications for TSA, an increase in trained surgeons for the procedures, and satisfactory long-term outcomes.^6^^,^^15^^,^^19^^,^^36^^,^^46^ Most prevalent indications for TSA are irreparable rotator cuff tears, cuff tear arthropathy, complex proximal humerus fractures, post-traumatic sequelae, and severe osteoarthritis.^12^^,^^18^^,^^34^^,^^38^

The aim of the procedure is to reduce pain and optimize shoulder function. Since TSA is increasingly performed on a younger and more active population, the ability to return to sports or work has become more critical.^13^^,^^31^ Several systematic reviews have been published regarding return to sports or work after shoulder arthroplasty in recent years.^1^^,^^7^^,^^22^^,^^28^^,^^32^^,^^43^ These studies described the rate, and in some cases the level of return to sports or work.

However, to our knowledge, there are no systematic reviews regarding the reasons for not returning to sports or work. This information is essential to interpret the return rate and to address potential issues and obstacles patients may experience returning to their activities. Whether patients return to sports or work depends on a wide variety of factors and may not always directly relate to the surgical procedure.^35^^,^^17^ Therefore, this study aims to assess the rate of return to sports or work and the reasons not to return to sports or work in patients undergoing TSA.

## Methods

The systematic review and meta-analysis were performed according to the Preferred Reporting Items for Systematic Reviews and Meta-Analyses (PRISMA) guidelines.^29^

### Search strategy

The search was performed in the Embase, Medline, Cochrane Central Register of Controlled Trials, and Web of Science Core Collection databases. Google Scholar was used as an additional search engine. The search was conducted with the assistance of an information specialist. Search strings and additional information can be found in the Supplementary Appendix S1. The final search was executed on the 23rd of April 2024.

### Study selection

All duplicate articles were removed. Studies reporting return to work or return to sport after anatomical or reverse shoulder arthroplasty with a minimum follow-up of 2 years and/or studies reporting the reasons for no return to sport or no return to work after anatomical or reverse shoulder arthroplasty were included. Exclusion criteria were reviews of the literature, expert opinions, nonclinical studies, case reports, letters to the editor, abstracts, revision arthroplasty, resurfacing arthroplasty or “ream and run” procedures, non-English/Dutch/German/French/Italian articles, studies not mentioning the kind of shoulder arthroplasty, studies not mentioning the follow-up, and studies not reacting to e-mail in case of missing data. Abstract and full-text screening was performed by two authors (W.P. and A.M.) using Rayyan software (Rayyan Systems, Cambridge, MA, USA).^30^ Any disagreement between the two authors was resolved by discussion and consensus.

### Quality of the studies

The quality of the included studies was evaluated using the Methodological Index for Non-Randomized Studies (MINORS) score.^41^ All studies were evaluated on the following items: a clearly stated aim, inclusion of consecutive patients, prospective data collection, endpoints appropriate to the aim of the study, unbiased assessment of the study endpoints, follow-up period appropriate to the aim of the study, loss to follow-up of less than 5%, and prospective calculation of the study size. Additional items for comparative studies were: adequate control group, contemporary group, baseline group equivalence, and adequate statistical analysis. Each item scored 0 (not reported), 1 (reported but poorly or inadequately done), or 2 (reported, well done, and adequate), with a maximum score of 16 and 24 for, respectively, noncomparative and comparative studies. Two authors (W.P. and A.M.) performed this evaluation and possible disagreement was resolved by discussion and consensus.

### Data extraction

A standardized form was used for data extraction in evaluating the studies. This form covered study characteristics (year, design, number of patients, and follow-up), patients' demographics (age, gender, arm dominance, underlying diagnosis, type of sport/work, preoperative level of sport/work), type of arthroplasty (aTSA or rTSA), and assessment of results (rate of return, reasons to not return, time to return, and postoperative level of sport/work). For return-to-sport or work analysis, only articles with a minimum follow-up of 2 years were included. Regarding reasons for no return, no minimum follow-up was set. The reasons to not return, extracted from the studies, were categorized and then summarized in bar graphs. Pain, restricted range of motion, instability, weakness, fear of injury of the shoulder, and missing confidence in the shoulder were considered as shoulder-related reasons, and all other reasons (such as loss of interest or nonshoulder-related health issues) were considered as not shoulder-related.

Data were analyzed using R version 4.2.1 (R Foundation for Statistical Computing, Vienna, Austria) and R Studio (R Studio, Posit, Boston, MA, USA). The primary endpoints were the return to sport and return to work rate after aTSA or rTSA. The I^2^ index was assessed to measure the heterogeneity of results within the included studies. Substantial heterogeneity was defined as I^2^>50%.^14^ Both a random- and fixed-effects model was used and reported for each subject. In case of substantial heterogeneity, the result of the random-effects model was considered the primary outcome; otherwise, a fixed-effects model was discussed. The rate of return was reported as a mean with a 95% confidence interval (CI).

## Results

### Study selection

**Figure 1:**
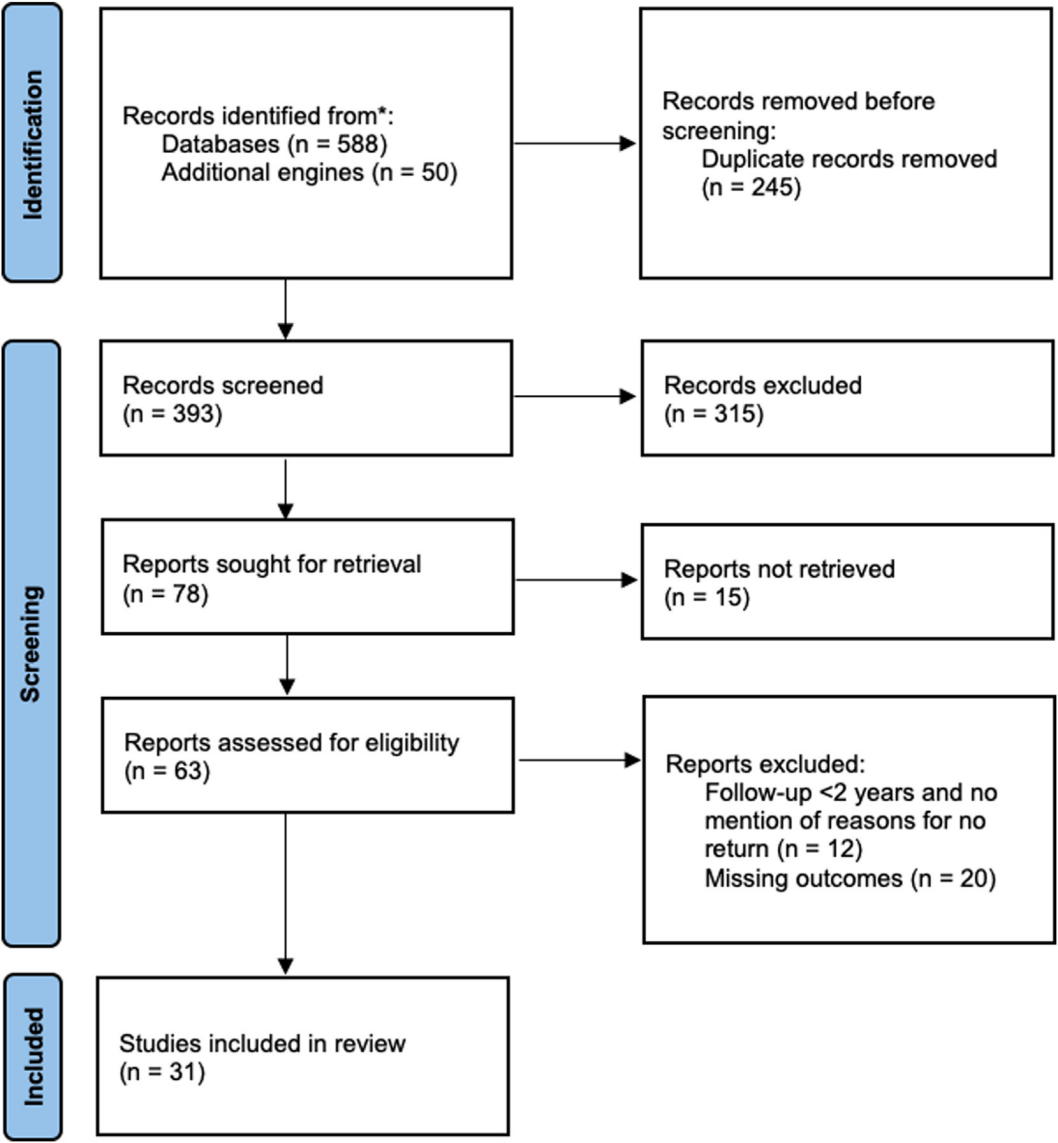
Flowchart of the study selection procedure.

Our search resulted in 393 articles. All articles were screened on title and abstract, after which 78 articles remained. After full-text screening, we included a total of 31 studies. Details about the study selection can be found in Figure 1.

### Methodological quality

A total of 16 studies were noncomparative and 11 studies were comparative. The mean (± standard deviation) MINORS score was 10.1 (±1.1) out of 16 for the 16 noncomparative studies and 16.2 (±1.9) out of 24 for the 10 comparative studies (Table I).

**Table I:** Study characteristics.

Study	Design	Level of evidence	Type of arthroplasty: n	Return to	Reasons mentioned (yes/no)	Minimum follow-up (yr)	Mean age	Mean follow-up (yr)	MINORS score
Ames, 2023^2^	Case series	IV	aTSA: 36	Sport	Yes	1	57.9	3.6	08/16
Bülhoff, 2015^3^	Case series	IV	aTSA: 154	Sport and work	Yes	2	72	6.2	10/16
Bülhoff, 2016^4^	Case series	IV	rTSA: 38	Sport and work	Yes	2	77.2	4.8	11/16
Cvetanovich, 2020^5^	Case series	IV	aTSA: 27	Sport and work	Yes	2	52.1	3.4	12/16
Fink Barnes, 2015^8^	Case series	IV	rTSA: 78	Sport	No	2	75.3	4.8	12/16
Garcia, 2019^11^	Case series	IV	aTSA: 30	Sport	No	2	53.6	5.8	17/24
Garcia, 2017^16^	Case series	IV	aTSA: 61	Sport	No	2	48.9	5.1	11/16
Garcia, 2016^9^	Case series	IV	rTSA: 40	Work	Yes	1	74.7	2.6	10/16
Garcia, 2015^8^	Case series	IV	rTSA: 76	Sport	Yes	1	74.8	2.6	10/16
Geyer, 2023^10^	Case series	IV	rTSA: 61	Sport	Yes	2	72.1	3.9	14/24
Godin, 2019^15^	Retrospective cohort study	III	rTSA: 110	Sport	No	2	68	3.6	19/24
Gowd, 2019^11^	Retrospective cohort study	III	aTSA: 28	Work	Yes	2	53.3	5.8	17/24
Hurwit, 2017^16^	Retrospective cohort study	III	rTSA: 40	Work	Yes	1	68.6	2.7	15/24
Kim, 2023^20^	Case control	III	rTSA: 44	Sport	Yes	2	72.9	3.4	10/16
Kolling, 2018^21^	Case series	IV	rTSA: 271	Sport	Yes	1	77.1	2.9	10/16
Kuijpers, 2023^23^	Case series	IV	aTSA: 44	Sport and work	Yes	1	57.6	4.6	07/16
Kusnezov, 2016^24^	Case series	IV	aTSA: 26	Work	Yes	1 and 2	45.8	3.4	10/16
Labriola, 2008^30^	Case series	IV	rTSA: 40	Sport	No	2	NR	NR	10/16
Lacheta, 2020^31^	Retrospective cohort study	III	rTSA: 33	Sport	No	2	63	2.9	15/24
Liu, 2020^26^	Retrospective cohort study	III	aTSA: 23	Work	Yes	2	61.7	5.2	17/24
Liu, 2018^27^	Case series	IV	aTSA: 52	Work	Yes	2	48.4	5.4	10/16
Mannava, 2017^38^	Case series	IV	aTSA: 186	Sport	No	2	64	3.7	10/16
McCarty, 2008^40^	Case series	IV	aTSA: 75	Sport	No	2	65.5	3.7	10/16
Morris, 2015^42^	Case control	III	rTSA: 14	Work	No	2	63.4	3.6	18/24
Pennington, 2023^33^	Case series	IV	rTSA: 106	Sport	No	2	72	NR	09/16
Schumann, 2010^37^	Case series	IV	aTSA: 55	Sport	Yes	1	66.2	2.3	09/16
Simovitch, 2015^39^	Case series	IV	rTSA: 67	Sport	No	2	73	3.6	07/16
Sinatro, 2016^40^	Retrospective cohort study	III	aTSA: 40	Sport	Yes	2	66.2	5.1	18/24
Tangtiphaiboontana, 2021^44^	Case series	IV	rTSA: 109	Sport	Yes	2	70	3.9	10/16
Wang, 2018^45^	Case series	IV	aTSA: 27, rTSA: 7	Sport	No	2	70.6	4.5	13/24
White, 2024^47^	Case series	IV	aTSA: 632	Sport	No	2	65.9	NR	08/16

*MINORS*, Methodological Index for Non-Randomized Studies; *aTSA*, anatomic total shoulder arthroplasty; *rTSA*, reverse total shoulder arthroplasty; *NR*, not reported.

### Study characteristics

Out of the 31 included studies, 23 were case series, 2 were a case-control study, and 6 were a retrospective cohort study. The level of evidence was III in 8 studies and IV in 23 studies. The return rate and, if reported, the reasons for no return were assessed in the studies with a minimum follow-up of 2 years. In the studies with a minimum follow-up of only 1 year, only the reasons were assessed. In total, 18 studies reported about the reasons why the patients did not return to their sport or work, whereas 13 studies did not report reasons for no return (Table I).

### Return to sport

#### aTSA

**Figure 2:**
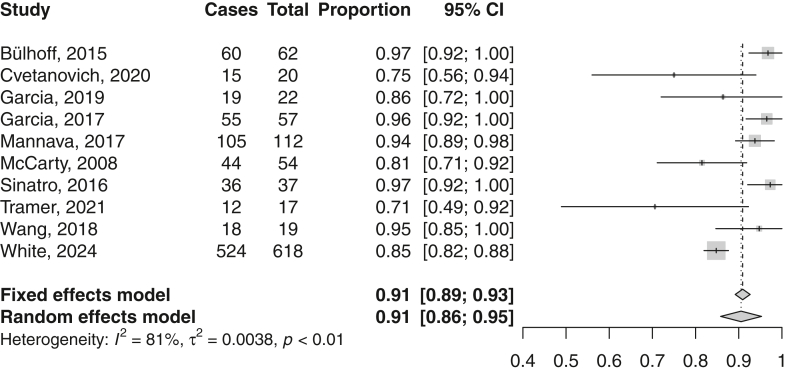
Forest plot on return to sport after anatomical total shoulder arthroplasty. *CI*, confidence interval.

The return rate to sport after aTSA was assessed in 10 studies. The mean (95% CI) return rate was 91% (86%-95%) (Fig. 2, Table II).

**Table II:** Return to sport after aTSA.

Study	Return rate (%)	Definition return	Mean time return (mo)	Level of return (%)
Bülhoff, 2015^3^	97	5 yr preop	NR	Same/higher level: 85
Cvetanovich, 2020^5^	75	3 yr preop	9.1	Higher level: 27
				Same level: 33
				Lower level: 40
Garcia, 2019^11^	86	NR	6.2	Same/higher level: 84
Garcia, 2017^16^	96	3 yr preop	6.7	Same/higher level: 82
Mannava, 2017^38^	94	NR	NR	Same/higher level: 70
McCarty, 2008^40^	81	NR	5.8	Higher level: 71
Sinatro, 2016^40^	97	NR	5.4	Same/higher level: 85
Wang, 2018^45^	95	NR	NR	NR
White, 2024^47^	85	NR	NR	NR

*aTSA*, anatomic total shoulder arthroplasty; *NR*, not reported.

**Figure 3:**
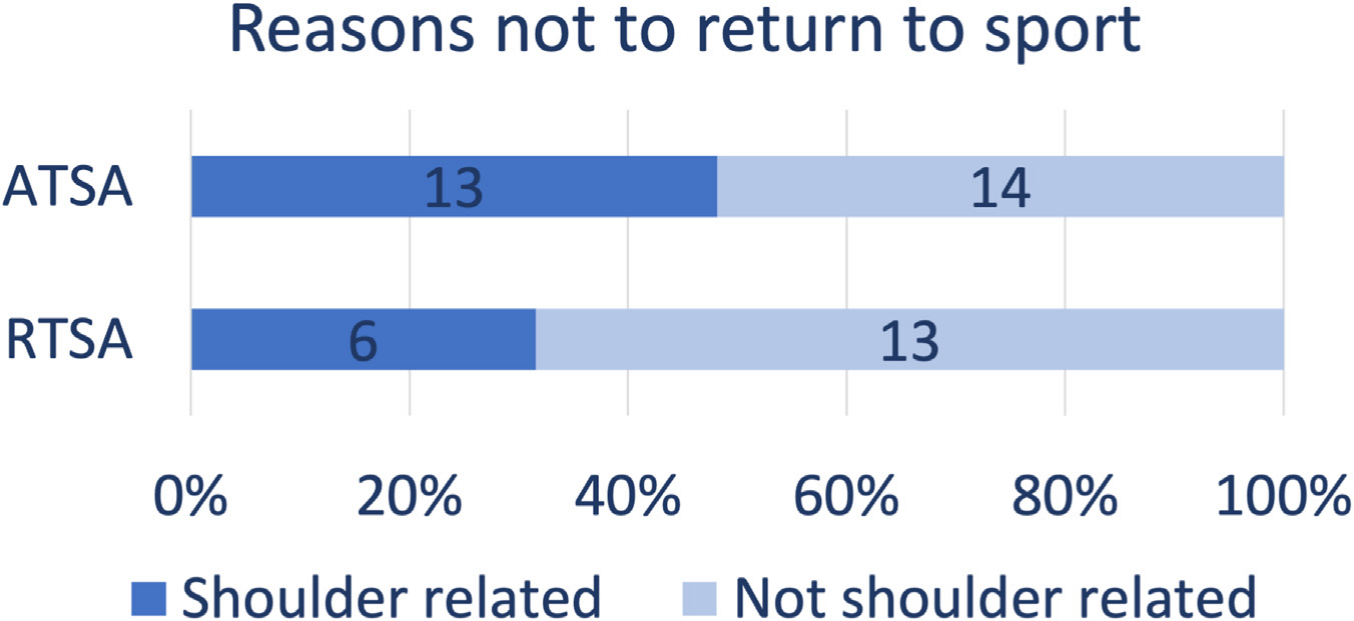
Summary of reasons not to return to sport after aTSA (anatomic total shoulder arthroplasty) and rTSA (reverse total shoulder arthroplasty).

Few studies reported the reasons why patients did not return to sport after aTSA. Cvetanovich et al mentioned that two of the five patients were unable to return because of shoulder-related reasons.^5^ The other three patients were unable to return because of comorbidities. Schumann et al stated that one patient did not return because of a shoulder-related reason, four patients did not return due to a not shoulder-related reason, and one patient quit sports because of combined reasons (shoulder and other).^37^ Ames et al reported 13 patients who did not return to weightlifting after aTSA, they cited activity modification to protect the arthroplasty (n = 6), shoulder pain (n = 2), COVID-19 (n = 2), health problems (n = 2), time (n = 1), and other (n = 2) as the causative factors.^2^ Sinatro et al only reported one patient who was unable to return to sport; the stated reason was pain.^40^ Bülhoff et al assessed the reasons of all patients that were not performing any sports at the final follow-up.^3^ Of the 45 patients of which the reasons were reported, 43 were not performing any sport 5 years prior to surgery. Eight patients were not participating because of shoulder-related problems, 23 reported other reasons, and 14 patients were no longer interested in participating in sports. Furthermore, 14 of the 60 patients who returned to sport had to change sports after surgery. In total, 13 patients did not return to sport after aTSA because of shoulder-related reasons, whereas 14 patients did not return because of not shoulder-related reasons (Fig. 3) .

#### rTSA

**Figure 4:**
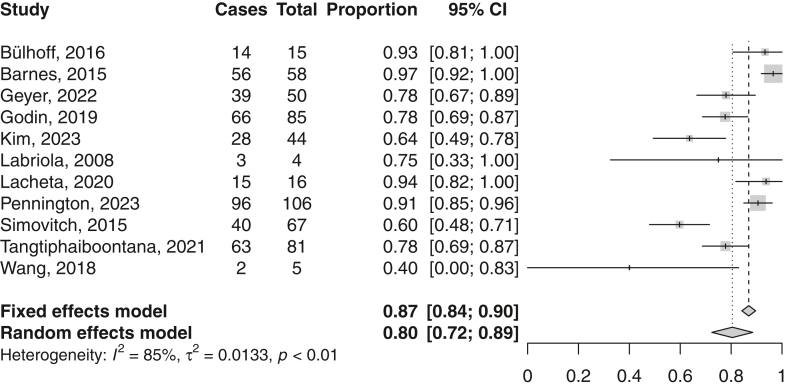
Forest plot on return to sport after reverse total shoulder arthroplasty. *CI*, confidence interval.

The return rate to sport after rTSA was assessed in 11 studies. The mean (95% CI) return rate was 80% (72%-89%) (Fig. 4, Table III).

**Table III:** Return to sport after rTSA.

Study	Return rate (%)	Definition return	Mean time return (mo)	Level of return (%)
Bülhoff, 2016^4^	93	5 yr preop	NR	Same/higher level: 86
Fink Barnes, 2015^8^	97	NR	NR	Same level: 58
Geyer, 2022^14^	78	NR	5.3	Higher level: 53
				Same level: 32%
				Lower level: 15
Godin, 2019^15^	78	NR	NR	NR
Kim, 2023^20^	64			Higher level: 46
				Same level: 21
				Lower level: 32
Labriola, 2008^30^	75	NR	NR	Lower level: 100
Lacheta, 2020^31^	94	NR	NR	NR
Pennington, 2023^33^	91	3 yr preop	NR	Same/higher level: 88
Simovitch, 2015^39^	60	NR	NR	Higher level: 30
				Same level: 65
				Lower level: 5
Tangtiphaiboontana, 2021^44^	78	NR	5.4	Same/higher level: 69,2
Wang, 2018^45^	40	NR	NR	NR

*rTSA*, reverse total shoulder arthroplasty; *NR*, not reported.

Several studies also mentioned the reasons why patients did not return to sport after rTSA. Bülhoff et al reported the reasons of all patients who were not performing any sports at the final follow-up.^3^ Of the seven patients of which the reasons were reported, six were not performing any sport 5 years prior to surgery. Three patients stated shoulder problems as the main reason, three stopped due to comorbidities, and one patient was no longer interested in sports. None of the patients who returned to sport were forced to change the kind of sport activity due to their shoulder replacement surgery. Garcia et al reported pain, problems due to the rTSA, and lack of interest as the most common reasons for discontinuation of sport.^8^ Geyer et al mentioned 13 patients, who had continued sports, had to give up over-head sports.^10^ The reasons for giving up those activities were fear in 21.1%, missing confidence in 31.6%, insufficient range of motion in 21.1%, pain in 15.8%, and the surgeon's recommendation in 10.5% of cases. Kolling et al stated that one patient gave up sports due to shoulder problems and two patients gave up sports because of reasons unrelated to the shoulder.^21^ Tangtiphaiboontana et al stated that restricted motion, fear of injury, and weakness were the most common reasons cited for inability to participate in sports.^44^ Kim et al reported the reason for 16 patients not to return to sport.^20^ Six had concerns about postoperative complication without any discomfort, five had postoperative pain or discomfort, three because of COVID-19 situation, and two because of a health problem other than shoulder. In total, six patients did not return to sport after aTSA because of shoulder-related reasons, whereas 13 patients did not return because of not shoulder-related reasons (Fig. 3).

### Return to work

#### aTSA

**Figure 5:**
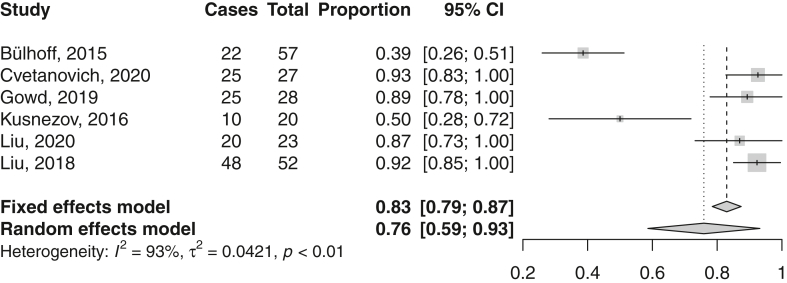
Forest plot on return to work after anatomical total shoulder arthroplasty. *CI*, confidence interval.

The return rate to work after aTSA was assessed in 6 studies. The mean (95% CI) return rate was 76% (59%-93%) (Fig. 5, Table IV).

**Table IV:** Return to work after aTSA.

Study	Return rate (%)	Definition return	Type of work	Mean time return (mo)	Level of return (%)
Bülhoff, 2015^3^	39	5 yr preop	NR	NR	NR
Cvetanovich, 2020^5^	93	NR	Heavy: 41%	3.7	Lower level: 24
			Medium: 33%		
			Light: 7%		
			Sedentary: 19%		
Gowd, 2019^11^	89	NR	Heavy: 14%	2	NR
			Moderate: 32%		
			Light: 18%		
			Sedentary: 36%		
Kusnezov, 2016^24^	50	Work at the time of surgery	Military	NR	NR
Liu, 2020^26^	87	3 yr preop	Heavy: 13%	1.3	Changed jobs: 0
			Light: 48%		
			Sedentary: 30%		
			NR: 9%		
Liu, 2018^27^	92	3 yr preop	Heavy: 21%	2.1	Changed jobs: 2
			Moderate: 33%		
			Light: 27%		
			Sedentary: 19%		

*aTSA*, anatomic total shoulder arthroplasty; *NR*, not reported.

**Figure 6:**
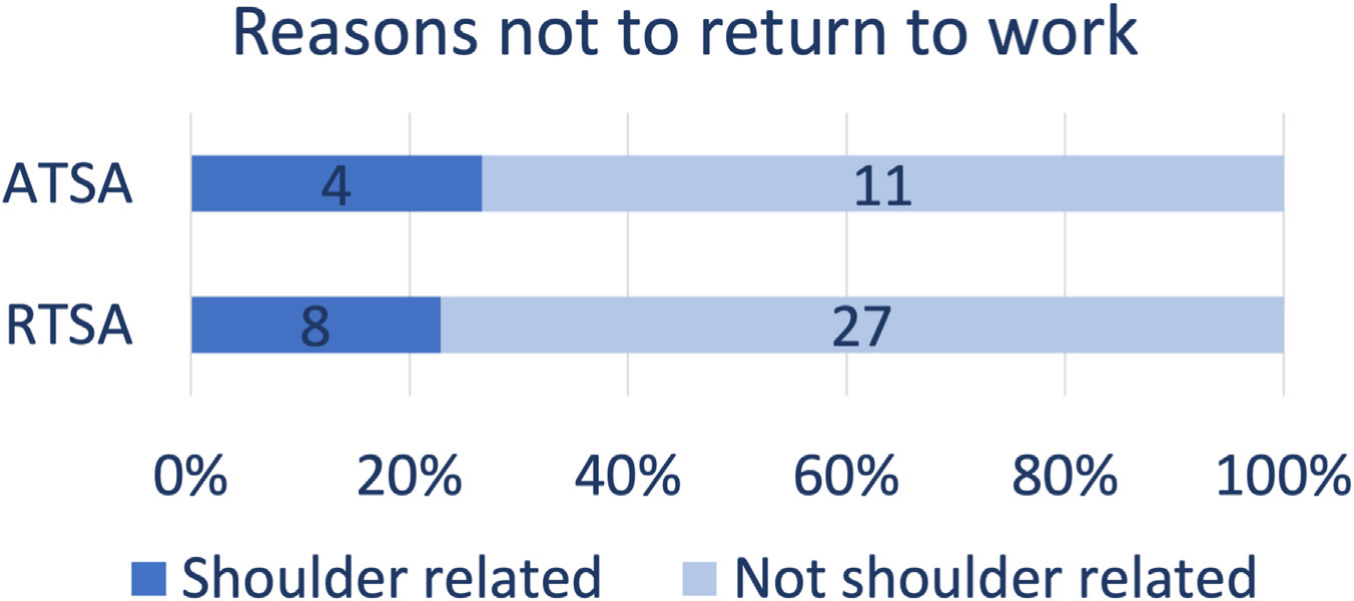
Summary of reasons not to return to work after aTSA (anatomic total shoulder arthroplasty) and rTSA (reverse total shoulder arthroplasty).

Few studies report reasons not to return to work after aTSA. Cvetanovich et al reported two patients who did not return to work after aTSA.^5^ One did not return because of reasons unrelated to the shoulder and one patient was unable to return because of back problems. Gowd et al mentioned one patient who was unable to return to his work as firefighter because of permanent restriction with overhead lifting after aTSA.^11^ The other patient was unable to return to his work as a construction worker because of reasons unrelated to his shoulder. Kusnezov et al reported nine patients were unable to return to military function because they were medically discharged from the military for persistent shoulder disability after aTSA.^24^ Liu et al stated that three patients did not return to work after aTSA.^26^ Two of the three patients had retired within the 3 years prior to surgery, one due to the shoulder and the other due to other medical reasons. The remaining patient retired due to nonspecified reasons. Liu et al reported that of the four patients who did not return to work, only the patient who underwent the postoperative conversion to reverse total shoulder for rotator cuff insufficiency retired specifically because of shoulder pain and limited range of motion.^27^ Two patients retired because of personal reasons unrelated to the shoulder, and the last patient retired because of other health-related issues. Kuijpers et al stated that one patient did not return to work because of postoperative complex regional pain syndrome, the other three did not return because of reasons unrelated to the shoulder.^23^ A total of four patients did not return to work after aTSA due to shoulder-related reasons, while eleven did not return due to reasons that were not shoulder-related (Fig. 6).

#### rTSA

**Figure 7:**
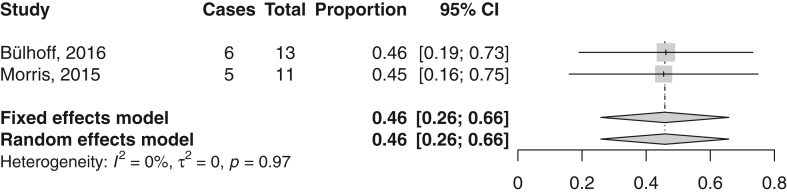
Forest plot on return to work after reverse total shoulder arthroplasty. *CI*, confidence interval.

The return rate to work after rTSA was assessed in 2 studies. The mean (95% CI) return rate was 46% (26%-66%) (Fig. 7, Table V).

**Table V:** Return to work after rTSA.

Study	Return rate (%)	Definition return	Type of work	Level of return
Bülhoff, 2016^4^	46	5 yr preop	NR	NR
Morris, 2015^42^	45	Employed work status	Sedentary/light: 7	No patients returned to heavy/strenuous work
			Heavy/strenuous: 4	

*rTSA*, reverse total shoulder arthroplasty; *NR*, not reported.

Bülhoff et al reported that four of the seven patients who stopped working after rTSA retired due to shoulder replacement surgery.^4^ Garcia et al stated that only two of the 14 patients retired because of shoulder-related reasons.^9^ The remaining 12 patients retired because of nonorthopedic causes. Hurwit et al mentioned the reasons of 14 patients who did not return to work.^16^ Two patients retired because of the shoulder after surgery, two retired preoperatively for medical reasons, and 10 retired because of other nonspecified reasons. Only one patient changed jobs after surgery, but this change was unrelated to her shoulder. In total, eight patients did not return to work after rTSA because of shoulder-related reasons, whereas 27 patients did not return because of not shoulder-related reasons (Fig. 6).

## Discussion

Our systematic review and meta-analysis aimed to provide insights into the rate of return to sports and work in patients with a TSA at a minimum follow-up of 2 years. Moreover, the reasons for not returning to these activities were assessed. The literature reporting on reasons not to return to sports or work is sparse and based on small cohorts. Our results show that reasons not related to the shoulder were much more common than shoulder-related reasons. The most common shoulder-related reasons are pain and limitations in range of motion.

Our meta-analysis showed that the rate of return to sports after aTSA was high, with a mean return rate of 91%. A few other meta-analyses assessed the return to sport after aTSA. Küffer et al reported a return rate of 77.4% after aTSA with a minimum follow-up of 0.5 years.^22^ Liu et al stated that 92.6% of the patients returned to sport after aTSA with a minimum follow-up of 0.5 years.^28^ Papalia et al showed a return rate of 90% without mentioning a minimum follow-up.^32^ These outcomes are similar to our outcome, although definitions of return to sport may differ between studies included in the reviews.

The rate of return to sports after rTSA was slightly lower than aTSA, with a mean return rate of 80%. While this rate is still favorable, it suggests that patients who undergo rTSA may face additional challenges in returning to sports compared to aTSA recipients. Several meta-analyses also assessed the return-to-sport rate after rTSA. Küffer et al reported a return rate of 71.2% after rTSA, and Liu et al reported a return rate of 74.9%, with a minimum follow-up of 0.5 years.^22^^,^^28^ Franceschetti et al showed a rate of return to sport after rTSA of 79% with a minimum 1-year follow-up.^7^ Papalia et al reported a return rate of 77%, and Aim et al reported 76.5%.^1^^,^^32^ Both studies did not mention a minimum follow-up. Similar to return to sport after aTSA, our study shows a relatively high return rate. This is arguably due to the minimum follow-up of 2 years. These results of both aTSA and rTSA suggest that the minimum follow-up of 2 years in our meta-analysis resulted in a slightly higher return rate, most likely because patients had more time to rehabilitate from the surgery. For future studies reporting return to activity after TSA, a minimum follow-up of at least 2 years should be considered and clearly reported.

Returning to work after TSA is another critical aspect of postoperative recovery, particularly for patients of working age. Our meta-analysis showed a mean return rate of 76% for aTSA and 46% for rTSA. These rates, while lower than those for returning to sports, still indicate a substantial number of patients who are able to resume their work duties after shoulder arthroplasty. To our knowledge, there is only one meta-analysis of the return to work rate after shoulder arthroplasty. Steinhaus et al assessed the return to work rate after aTSA and rTSA with a minimum follow-up of 1 year.^43^ The return rate after aTSA was 63.4% and after rTSA 61.5%. The minimum follow-up of 2 years in our study may result in a higher rate of return. On the other hand, most patients undergoing shoulder arthroplasty are approaching retirement age, decreasing the probability of returning to work.

Reasons for not returning are essential for interpreting the return rate. However, most studies on return to sport and work after shoulder arthroplasty do not mention the reasons for no return. Furthermore, if reasons are mentioned, the reasons are often not specified. Nevertheless, we have assessed all literature regarding reasons to not return to sport or work after shoulder arthroplasty. Remarkably, most reasons for not returning to sport or work after aTSA or rTSA are not shoulder-related. When interpreting results regarding return rate, this must be taken into consideration. This may also aid orthopedic care providers when informing patients about the probability of returning to sport or work after shoulder arthroplasty. Therefore, it is important to mention the reasons for no return in future studies regarding return to sport or work. Specific reasons should be mentioned because not all reasons are strictly shoulder-related or not-shoulder-related. Patients can have multiple reasons not to return, and reasons can be both partly shoulder-related and partly not shoulder-related. Not shoulder-related reasons to not return to sport were mainly lack of interest, comorbidities, and personal factors. Most patients who did not return to work for reasons unrelated to the shoulder were already about to retire. Few studies report these results or are incomplete in their reporting. The results of this review argue that reasons not to return to activity should be reported in detail with a minimum follow-up of 2 years.

Nevertheless, it is important to note that while many patients successfully return to sports and work after shoulder arthroplasty, a portion of the patient population still faces challenges in resuming these activities. Shoulder-related reasons why patients did not return to sport after shoulder arthroplasty were pain, limited range of motion, fear of injury, and lack of confidence. Patients not returning because of fear of injury or lack of confidence may benefit from psychological guidance. Therefore, it is important to assess the reasons per patient as a healthcare provider. Shoulder-related reasons to not return to work were pain and restricted range of motion.

To our knowledge, no systematic reviews about shoulder arthroplasty assessed the reasons why patients did not return to sport or work. A few systematic reviews have assessed the reasons why patients did not return to sport after hip or knee arthroplasty. Lester et al stated surgeon instruction, preservation of implant, pain, and apprehension as reasons patients did not return to sport after knee arthroplasty.^25^ Sowers et al stated that patients did not return to sport after hip arthroplasty because of surgeon advice.^42^ In contrast to our findings, in the lower extremity studies, no reasons for not returning to sport were reported that were unrelated to orthopedic pathologies.

The reasons for not returning to sports or work can be multifactorial, and they underscore the need for a comprehensive approach to patient care. Healthcare providers should engage in thorough preoperative counseling to manage patient expectations and provide a realistic understanding of the potential postoperative outcomes. Additionally, a detailed assessment of comorbidities and individual patient factors is essential to identify those at greater risk of not returning to sports or work.

Based on our findings, several criteria for reporting return to sports or work after shoulder arthroplasty can be proposed. We believe that future studies should consider adhering to the following criteria:

- A minimum follow-up of 2 years.
- A detailed report of preoperative activities, including type of work (manual or sedentary), type of sport (contact, overhead, involving the upper body, or lower body only), and level of sport (recreative, competitive, or professional).
- A report of postoperative instructions that patients received, including functional limitations advised by the healthcare provider.
- A detailed report of postoperative activities, including return to the same type and level of activities.
- A report of reasons for not returning to work or sport, including whether the reason is shoulder-related or unrelated. The presence of kinesiophobia and fear of injury should be specifically assessed. It also should be considered that multiple reasons may apply.
- A clear description of an active sports performer or worker. What period of time of participation in sport or work before surgery is considered active?
- A clear definition of when a patient has returned to work or sport. What level of sport is considered as returned? What frequency of work is considered as returned?

### Limitations

Our study has several limitations that warrant consideration. First, there is inherent heterogeneity among the included studies, which may stem from variations in study design, patient populations, surgical techniques, and definition of return to sport or work. Heterogeneity was reported and taken into account when interpreting the results of the meta-analysis. There was little difference between the random- and fixed-effect models.

Second, the quality of the included studies, as assessed by the MINORS score, ranged from moderate to high. While this reflects the overall quality of the evidence, it is important to acknowledge that biases and limitations inherent to observational studies may have influenced the results.

Finally, the reasons for not returning to sports or work were not consistently reported in all the included studies. Only a few studies reported the reasons for not returning. This limited our ability to provide a comprehensive analysis of the factors contributing to non-return. Furthermore, it underscores the need for mentioning reasons for no return in future research about return to sport or work.

## Conclusion

A high return to sport can be expected after TSA. The rate of return to work after aTSA is high; this is in contrast to patients with an rTSA who are less likely to return to work. In the current literature, reporting on reasons not to return is limited. Interestingly, most reasons not to return to sport or work after shoulder arthroplasty are not shoulder related, suggesting that a lack in return to sport or work is a poor indicator for the result of shoulder arthroplasty. This suggests that the value of return to work or sports rates as a measure of the success of surgery is limited. This should be considered when interpreting studies reporting return to work or sports and can be used by orthopedic care providers to set expectations.

## Disclaimers:

Funding: The authors declare that no source of support in the form of grants, equipment, or other items was received for this study.

Conflicts of interest: The authors, their immediate families, and any research foundation with which they are affiliated have not received any financial payments or other benefits from any commercial entity related to the subject of this article.
