# Supplementary material for: Why do patients not return to sports or work after anatomical or reverse total shoulder arthroplasty? A systematic review and meta-analysis
Source: JSES Int. 2025 Jun 7;9(5):1713–22. doi: 10.1016/j.jseint.2025.05.028 (PMC12490628; doi:10.1016/j.jseint.2025.05.028)
Supplement: Supplementary Appendix S1 [file mmc1.pdf]

Database searched	Platform	Years of coverage	Records	Records after duplicates removed
Embase	Embase.com	1971 - Present	305	304
Medline ALL	Ovid	1946 - Present	168	63
Web of Science Core Collection*	Web of Knowledge	1975 - Present	112	19
Cochrane Central Register of Controlled Trials	Wiley	1992 - Present	3	3
Additional Search Engines: Google Scholar			50	4
**Total**			**638**	**393**

*Science Citation Index Expanded (1975-present) ; Social Sciences Citation Index (1975-present) ; Arts & Humanities Citation Index (1975-present) ; Conference Proceedings Citation Index- Science (1990-present) ; Conference Proceedings Citation Index- Social Science & Humanities (1990-present) ; Emerging Sources Citation Index (2005-present)

Embase.com

('shoulder arthroplasty'/exp OR (arthroplasty/de AND ('shoulder'/exp OR 'shoulder injury'/de)) OR (((shoulder*) NEAR/3 (arthroplast* OR replacement*))):ab,ti,kw) AND ('return to work'/exp OR 'return to sport'/exp OR 'sport'/exp OR (((activit* OR return* OR participat*) NEAR/9 (work* OR sport*))):ab,ti,kw)

Medline Ovid

(Arthroplasty, Replacement, Shoulder/ OR (Arthroplasty/ AND (Shoulder/ OR exp Shoulder Injuries/)) OR (((shoulder*) ADJ3 (arthroplast* OR replacement*))).ab,ti,kf.) AND (Return to Work/ OR Return to Sport/ OR exp Sports/ OR (((activit* OR return* OR participat*) ADJ9 (work* OR sport*))).ab,ti,kf.)

Web of Science

TS=(((((shoulder*) NEAR/2 (arthroplast* OR replacement*)))) AND ((((activit* OR return* OR participat*) NEAR/9 (work* OR sport*)))))

Cochrane Central

((((shoulder*) NEAR/3 (arthroplast* OR replacement*))):ab,ti,kw) AND ((((activit* OR return* OR participat*) NEAR/9 (work* OR sport*))):ab,ti,kw)

Google Scholar top 50 relevant records

"shoulder arthroplasty|arthroplasties|replacement|replacements" "return*work|sport|sports"
